# Smooth muscle cell-specific *Tgfbr1* deficiency promotes aortic aneurysm formation by stimulating multiple signaling events

**DOI:** 10.1038/srep35444

**Published:** 2016-10-14

**Authors:** Pu Yang, Bradley M. Schmit, Chunhua Fu, Kenneth DeSart, S. Paul Oh, Scott A. Berceli, Zhihua Jiang

**Affiliations:** 1 Division of Vascular Surgery and Endovascular Therapy, University of Florida College of Medicine, Gainesville, FL 32610, United States; 2 Department of Surgery, Central South University Xiangya Hospital, Changsha, Hunan, P.R. China; 3 Department of Physiology and Functional Genomics, University of Florida College of Medicine, Gainesville, FL 32610, United States; 4 Malcom Randall VA Medical Center, Gainesville, FL 32608, United States

## Abstract

Transforming growth factor (TGF)-β signaling disorder has emerged as a common molecular signature for aortic aneurysm development. The timing of postnatal maturation plays a key role in dictating the biological outcome of TGF-β signaling disorders in the aortic wall. In this study, we investigated the impact of deficiency of TGFβ receptors on the structural homeostasis of mature aortas. We used an inducible Cre-loxP system driven by a *Myh11* promoter to delete *Tgfbr1, Tgfbr2,* or both in smooth muscle cells (SMCs) of adult mice. TGFBR1 deficiency resulted in rapid and severe aneurysmal degeneration, with 100% penetrance of ascending thoracic aortas, whereas TGFBR2 deletion only caused mild aortic pathology with low (26%) lesion prevalence. Removal of TGFBR2 attenuated the aortic pathology caused by TGFBR1 deletion and correlated with a reduction of early ERK phosphorylation. In addition, the production of angiotensin (Ang)-converting enzyme was upregulated in TGFBR1 deficient aortas at the early stage of aneurysmal degeneration. Inhibition of ERK phosphorylation or blockade of AngII type I receptor AT1R prevented aneurysmal degeneration of TGFBR1 deficient aortas. In conclusion, loss of SMC-*Tgfbr1* triggers multiple deleterious pathways, including abnormal TGFBR2, ERK, and AngII/AT1R signals that disrupt aortic wall homeostasis to cause aortic aneurysm formation.

Recent clinical investigations have linked transforming growth factor (TGF) -β signaling disorders to the development of aortic aneurysms. When mutated, genes encoding TGFβ signaling components, such as the ligand, receptors, and intracellular mediators, can cause the formation of aortic aneurysms, leading to Loeys-Dietz syndrome (LDS)^1^,^2^. Familial thoracic aortic aneurysm and dissection (TAAD) can also be caused by mutations of TGFβ receptors (e.g. TGFBR1^3^ and TGFBR2^4^). In LDS patients, aortic tissue, which harbors defective TGFβ signaling components, paradoxically, but consistently, shows enhanced TGFβ activity, as evidenced by increased SMAD2 phosphorylation and the expression of CTGF^2^,^5^,^6^. These observations have led to a theory that TGFβ hyperactivity accounts for the aortic pathology^7^. Accordingly, loss-of-function mutation of the inhibitory TGFβ signaling component SKI predisposes patients to aortic aneurysm formation^8^. This notion is further supported by findings that mutations of genes not specific to TGFβ signaling transduction (e.g., *ACTA2* and *MYH11*) also augment TGFβ signaling in the aortic wall, causing familial thoracic aortic aneurysm and dissection (TAAD)^9^. Further evidence for the causative role of TGFβ comes from studies of Marfan syndrome (MFS), in which affected organs contains abnormally high levels of active TGFβ^10^, and serological removal of TGFβ effectively prevents aortic dilation and elastic fiber fragmentation^11^.

However, evidence conflicting with the theory of TGFβ hyperactivity exists in the literature. For example, longitudinal studies of mouse models of LDS show that enhanced TGFβ signaling, canonical or non-canonical, is not evident until aortic pathology has advanced a relatively late stage^12^. It is also noteworthy that direct evidence supporting TGFβ hyperactivity as the mechanism underlying genetic TGFβ defects remains lacking, whereas haploinsufficiency of TGFB2^13^, TGFB3^2^, and SMAD3^14^ causes aortic aneurysm formation. In addition, a series of studies from Gomez *et al*. suggests that the expression of *SMAD2* is upregulated in medial smooth muscle cells (SMCs) due to epigenetic modification. The elevated production of total SMAD contributes to the phenomenon of “TGFβ paradox” in a mechanism independent of the local concentration of TGFβ proteins^15^,^16^,^17^,^18^. These lines of evidence have raised a critical question about the exact role of TGFβ in the development of aortic pathology that is linked to genetic TGFβ signaling defects.

TGFβ is a potent growth factor that participates in vascular development via regulating cell differentiation and matrix metabolism^19^. Germline deletion of TGFβ signaling components such as receptors and ligands is thus embryonically lethal due to vascular defects of the yolk sac^20^. Further studies for the function of TGFβ in individual cell-groups demonstrate that deficiency of SMC-specific TGFβ induces persistent truncus arteriosus^21^,^22^. While the importance of TGFβ in vascular development is widely-recognized, its physiological role in established vasculature, particularly the aortic wall, is less-well understood. Patients suffering from genetic TGFβ disorders are usually born with a normal aorta with aneurysms developing in the postnatal life. Although the underlying mechanisms could be multifold, it is possible that the genetic mutations induce the aortic pathology through interrupting the physiological function of TGFβ. This hypothesis is consistent with the finding that a basal level of TGFβ protects aortas from chemical-induced aneurysm formation^23^. We have recently reported that the ablation of SMC-specific *TGFBR1* causes aortic aneurysms and dissections^24^. Since *TGFBR1* can form a receptor complex with multiple type II TGFβ receptor members^25^, it was unclear whether the phenotype resulting from the loss of SMC TGFBR1 is driven by the TGFBRII signal. Therefore, this current study evaluated the role of TGFBR2 in the phenotypic expression of the aortas deficient in TGFBR1. While our study was being completed, two alternate studies reported that the deletion of SMC-specific *Tgfbr2* induces aortic aneurysmal degeneration with complete penetrance in immature but not adult mice^26^,^27^. The current study, however, using a series of genetic experiments in mice, investigated the functional relationship between TGFβ receptors and aorta homeostasis. Results from our study show that SMC-*Tgfbr1* deletion promoted aortic aneurysm formation in a manner partially dependent on *Tgfbr2* in adult mice. The aneurysmal degeneration could be rescued by inhibition of phosphorylation of extracellular signal-regulated kinase (ERK) or blockade of angiotensin type I receptor (AT1R), indicating that multiple pathways deleterious to the aortic wall are activated following SMC-*Tgfbr1* deletion.

## Results

### SMC-specific deletion of the TGFβ receptor members

We used an inducible Cre-loxP system driven by a *Myh11* promoter^26^ to delete SMC-specific *Tgfbr1* (*Tgfbr1*^*iko*^), *Tgfbr2* (*Tgfbr2*^*iko*^), or both *Tgfbr1* and *Tgfbr2* (*Tgfbr1*^*iko*^*.Tgfbr2*^*iko*^). To confirm efficient gene-deletion in aortas, we monitored the recombination-events with a *Gt(ROSA)26sor* (*R26R*) reporter strain (*n* = 3–6 for each genotype). Shown in Supplementary Figure 1A are representative images of x-gal staining of the ascending aortic segments (ATAs) collected on the following day of the last dose of tamoxifen injection. Tamoxifen induced robust and similar degree of recombination-events in SMCs of *Tgfbr1*^*iko*^, *Tgfbr2*^*iko*^, and *Tgfbr1*^*iko*^*.Tgfbr2*^*iko*^ ATAs, as evidenced by positive x-gal staining of vast majority of cells throughout the entire medial layer. Genotyping assays on explanted SMCs confirmed the null allele for *Tgfbr1* and *Tgfbr2* (Supplementary Figure 1B). We further validated the deletion of these receptors with functional assays. In contrast to *Tgfbr1*^*f/f*^ controls, *Tgfbr1*^*iko*^ and *Tgfbr2*^*iko*^ SMCs showed no pSMAD2 expression or upregulation of TGFβ -responsive genes (e.g., *Ctgf*, c*ol1a2*, and *Eln*) after TGFβ stimulation (Supplementary Figure 2A,B). Activation of ERK by TGFβ was also abolished in these SMCs (Supplementary Figure 2C). Therefore, the system induced efficient deletion of the TGFβ receptors *in vivo*.

### Deletion of SMC-specific *Tgfbr1* in adult mice rapidly causes aneurysmal degeneration of the thoracic aorta

**Figure 1:**
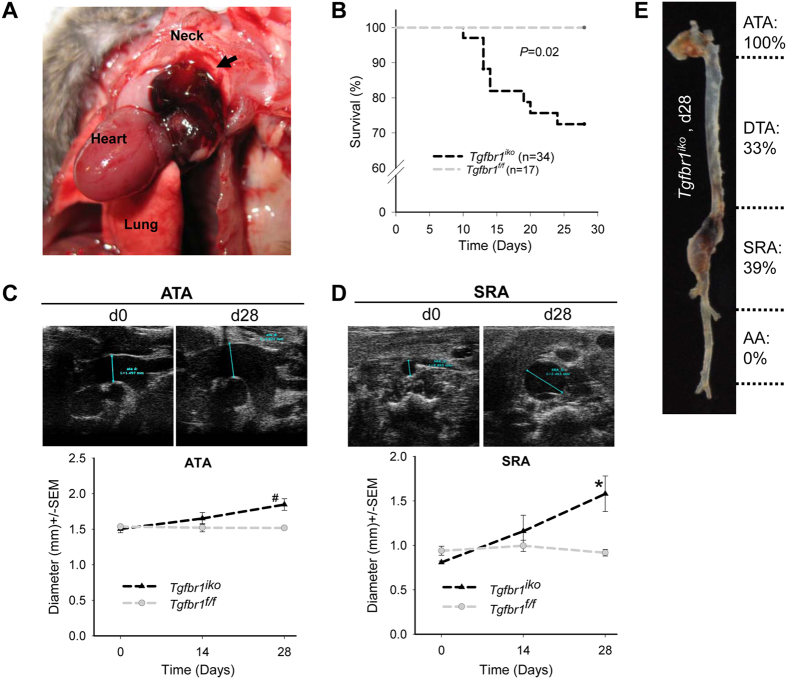
*Tgfbr1*^*iko*^ rapidly results in aortic rupture and aneurysmal degeneration. (**A**) A gross specimen of a *Tgfbr1*^*iko*^ aorta showing a hematoma (arrow) resulting from rupture of the ascending aorta (ATA) on day (d) 13. (**B**) Kaplan Meier survival analysis of *Tgfbr1*^*iko*^ (*n* = 34) and *Tgfbr1*^*f/f*^ (*n* = 17) animals. (**C**,**D**) Ultrasound imaging of *Tgfbr1*^*f/f*^ (*n* = 5) and *Tgfbr1*^*iko*^ (*n* = 9) aortas at sites of ATA (**C**) and suprarenal (SRA) (**D**) segments. Significance of genotype- and time-dependent differences was evaluated using two-way repeated measurement ANOVA. ^#^*P* = 0.003, *P = 0.027. (**E**) Incidence of grossly evident pathologies at various anatomic locations of surviving*Tgfbr1*^*iko*^ mice (*n* = 24). DTA: descending aorta; AA: infrarenal aorta. Note that nearly 40% of the aortas had pathologies at multiple locations.

With the validated Cre-loxP system, we induced *Tgfbr1*^*iko*^ by tamoxifen injection in male mice at 9–13 weeks of age (*n* = 34) and injected age- and sex-matched *Tgfbr1*^*f/f*^ mice with tamoxifen (*n* = 17) as a control. In *Tgfbr1*^*iko*^ animals, aortic rupture was noted as early as 10 days after the first dose of tamoxifen (Fig. 1A). By day 28 (d28), 29% (10/34) of *Tgfbr1*^*iko*^ animals died from an aortic rupture located in ATA (2), proximal descending (DTA, 2), or suprarenal (SRA, 6) segments, but all *Tgfbr1*^*f/f*^ controls survived (Fig. 1B). Surviving *Tgfbr1*^*iko*^ animals experienced progressive aortic enlargement. As determined by ultrasound scanning of a subset of age-matched (11 weeks) *Tgfbr1*^*iko*^ (*n* = 9) and *Tgfbr1*^*f/f*^ (*n* = 5) mice on d28, *Tgfbr1*^*iko*^ mice exhibited a 23% and 78% increase in diameter in the ATA and suprarenal (SRA) aortic segments, respectively, whereas *Tgfbr1*^*f/f*^ controls did not present significant changes (Fig. 1C,D). When gross examination was performed on d28, all *Tgfbr1*^*iko*^ aortas displayed one or more evident pathologies, which were manifested by fusiform dilation, intramural hematoma, intimal/medial tear, and contained rupture (Fig. 1E, Supplementary Figure 3). The prevalence of aortic pathologies varied depending on the aortic region. Of the 24 surviving *Tgfbr1*^*iko*^ animals, aortic lesions were noted in 100% of ATA segments, 33% of DTA segments, and 39% of SRA segments, but not in any infrarenal (AA) segments (Fig. 1E).

**Figure 2:**
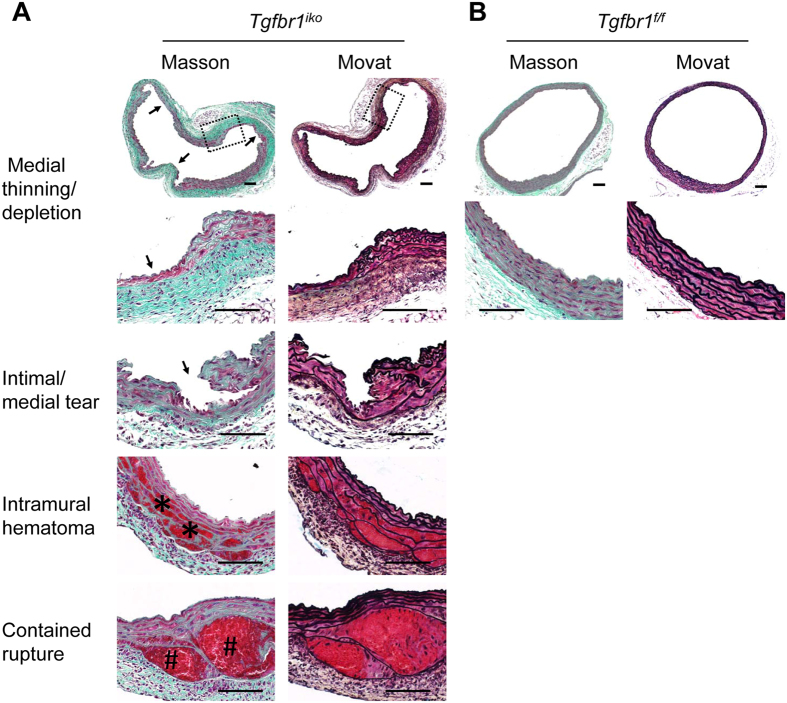
*Tgfbr1*^*iko*^ recapitulates a spectrum of pathologies that are typical of aortic aneurysmal degeneration. (**A**) Masson’s (left column) and Movat’s (right column) staining of cross sections of *Tgfbr1*^*iko*^ ATAs that were collected at d28. Arrows and symbols point to the pathology specified on the side of the columns. The boxed area is amplified in the lower panels to show structural details of the medial thinning. (**B**) Histology representative of *Tgfbr1*^*f/f*^ controls. Scale bars: 100 μm.

Next, we intensively characterized the progression of aortic pathologies over time. Because of the complete penetrance in ATA segments, we focused our evaluation on this location throughout the study. To assess the early histological changes, *Tgfbr1*^*iko*^ ATAs were harvested at d2 (n = 6), d5 (n = 7), and d10 (n = 12) and *Tgfbr1*^*f/f*^ ATAs collected at d10 (n = 7) served as controls. Elastic fiber fragmentation was detected as early as 10 days after the first dose of tamoxifen in *Tgfbr1*^*iko*^ ATAs (Supplementary Figure 4A). Medial thickness of these ATAs did not change significantly and remained similar to that of the controls at this time point (Supplementary Figure 4B). Intimal/medial tears were caught randomly in cross-sections of d10 ATAs. To evaluate the distribution of this pathology in the entire ATA segments, we injected Evans Blue to animals prior to tissue collection to highlight areas with intimal/medial tears and examined the specimens with *en face* microcopy using the same protocol as previously described^24^. Specimens (*n* = 3 per genotype) were collected on d13, the time point around which *Tgfbr1*^*iko*^ aortas frequently rupture (Fig. 1B). As shown in Supplementary Figure 4C, scattered and randomly distributed blue spots were observed on the luminal surface of *Tgfbr1*^*iko*^ ATAs. Scanning electron microscopy (SEM) and histological evaluations confirmed that these focal lesions were intimal/medial tears that often extended deep into the media (Supplementary Figure 4C). Pathologies at a more advanced stage (d28) were characterized by randomly distributed areas of medial thinning or complete depletion (16/24, 67%), intimal-medial tears (17/24, 71%), intramural hematoma (12/24, 50%), and contained rupture (3/24, 13%). Intense adventitial fibrosis was evident in all *Tgfbr1*^*iko*^ ATA segments (Fig. 2A,B). The pathological features displayed by the SRA segments were essentially the same as those presented by the ATA specimens (Supplementary Figure 5). False lumen formation, which was rarely detected in the ATA region, was occasionally noted for SRA specimens (Supplementary Figure 5E), indicating that animals with acute SRA dissections had a higher chance to survive than those with acute ATA dissections.

### Deletion of SMC-specific *Tgfbr2* is less deleterious to the aortic wall than deletion of *Tgfbr1*

**Figure 3:**
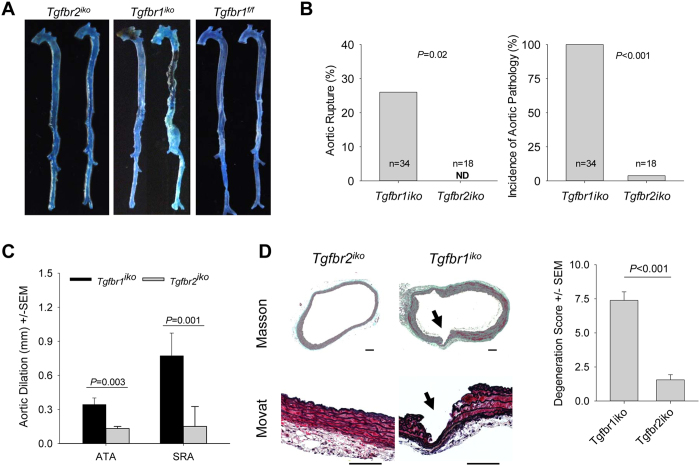
*Tgfbr2*^*iko*^ only causes mild aortic pathology with low phenotypic penetrance in 28 days. (**A**) Gross image of aortic specimens with the indicated genotypes. Dark patches appearing in *Tgfbr1*^*iko*^ aortas reflect intramural hematoma. (**B**) Surgical evaluation of aortic pathology (aneurysm, intimal/medial tears, intramural hematoma, contained or free rupture). Fisher’s exact test was performed for data analysis. (**C**) Changes in aortic diameter at the indicated locations in *Tgfbr1*^*iko*^ (*n* = 9) and *Tgfbr2*^*iko*^ (*n* = 9) mice. Differences between groups were evaluated using unpaired *t*-tests. (**D**) Left: Histological evaluation of aortic structural degeneration. Images show typical histology of *Tgfbr2*^*iko*^ and *Tgfbr1*^*iko*^ ATAs. Arrows indicate a deep intimal/medial tear. Right: Degeneration score for *Tgfbr1*^*iko*^ (*n* = 24) and *Tgfbr2*^*iko*^ (*n* = 18) ATA segments. Data were analyzed using the unpaired *t*-test. Scale bars: 100 μm.

TGFβ is thought to signal through *Tgfbr2* and then *Tgfbr1* receptor subunits^28^, suggesting that *Tgfbr2*^*iko*^ would lead to the same aortic phenotype as *Tgfbr1*^*iko*^. To test this hypothesis, we induced *Tgfbr2*^*iko*^ in male mice (*n* = 18) at 9 to 13 weeks of age and evaluated aortic phenotypes at d28. Surprisingly, all *Tgfbr2*^*iko*^ mice survived for at least 28 days. On gross examination, only one *Tgfbr2*^*iko*^ mouse exhibited a fusiform aneurysm in the SRA segment, whereas all other mice displayed aortic morphology that was grossly undistinguishable from that of *Tgfbr1*^*f/f*^ controls (Fig. 3A). Aortic rupture was not observed in any of the *Tgfbr2*^*iko*^ mice but occurred in 26% of *Tgfbr1*^*iko*^ mice. The incidence of gross aortic pathology was 6% in *Tgfbr2*^*iko*^ animals but 100% in *Tgfbr1*^*iko*^ animals (Fig. 3B). When we performed ultrasound scanning for a subset of age-matched *Tgfbr2*^*iko*^ animals (*n* = 9), *Tgfbr2*^*iko*^ aortas showed only a modest increase in diameter over 28 days that was significantly less than that observed for *Tgfbr1*^*iko*^ aortas (Fig. 3C). Pathologies detected in *Tgbr2*^*iko*^ ATA segments at the histological level were limited to elastic fiber breaks (7/18, 39%) and shallow intimal/medial tears (3/18, 17%). We developed a scoring system to quantify these pathologies (See details in Materials and Methods and Supplementary Figure 6A–C) and found that *Tgfbr2*^*iko*^ resulted in a significantly lower degree of aneurysmal degeneration than *Tgfbr1*^*iko*^ (Fig. 3D). These *in vivo* observations suggest that the functions of *Tgfbr2* and *Tgfbr1* receptors in SMCs do not completely overlap, with *Tgfbr1* being more critical than *Tgfbr2* to the maintenance of aortic wall homeostasis in adult animals. An alternative explanation, however, is that the distinct phenotypes result from abnormal *Tgfbr2* signal triggered by *Tgfbr1* deficiency.

### Deleterious effect of SMC-specific *Tgfbr1* deficiency relies largely on *Tgfbr2*

**Figure 4:**
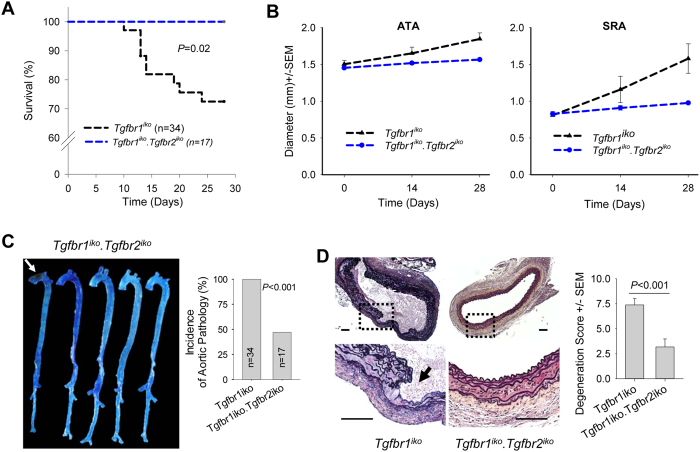
Removal of *Tgfbr2* attenuates the aortic phenotype induced by *Tgfbr1*^*iko*^. (**A**) Kaplan Meier survival analysis shows that removal of *Tgfbr2* completely rescued aortic rupture induced by *Tgfbr1*^*iko*^. (**B**) Changes in the aortic diameter of *Tgfbr1*^*iko*^ (*n* = 9) and double *Tgfbr1*^*iko*^.*Tgfbr2*^*iko*^ (*n* = 11) aortas at the indicated locations, as revealed by ultrasound scanning. Data were evaluated using two-way repeated measurement ANOVA. ATAs: Genotype, *P* = 0.04; Time, *P* = 0.03. SRAs: Genotype, *P* = 0.02; Time, *P* < 0.001. (**C**) Gross examination. Arrow points to an intramural hematoma (d28). *P* < 0.001, Fisher’s exact test. (**D**) Left: Histological evaluation of ATA segments (d28). Arrow indicates a deep intimal/medial tear. Right: Degeneration score for *Tgfbr1*^*iko*^ (*n* = 24) and *Tgfbr1*^*iko*^.*Tgfbr2*^*iko*^ (*n* = 17) ATA segments. Data were analyzed using the unpaired *t*-test. Scale bars: 100 μm.

The distinct effects of *Tgfbr1* and *Tgfbr2* deletion on aortic wall homeostasis suggest different underlying mechanisms. If the abnormal *Tgfbr2* signaling accounts for the aortic disease, then removing *Tgfbr2* should prevent aneurysmal degeneration of *Tgfbr1*^*iko*^ aortas. We therefore generated the *Tgfbr1*^*iko*^*.Tgfbr2*^*iko*^ strain to test this hypothesis. Male *Tgfbr1*^*iko*^*.Tgfbr2*^*iko*^ mice (*n* = 17) at 9–11 weeks of age were treated with tamoxifen and followed up for 28 days. Removal of *Tgfbr2* fully rescued aortic rupture induced by *Tgfbr1*^*iko*^ (Fig. 4A). Compared with *Tgfbr1*^*iko*^, *Tgfbr1*^*iko*^*.Tgfbr2*^*iko*^ significantly attenuated aortic dilation at ATA (23% vs. 8%) and SRA (78% vs. 8%) segments (Fig. 4B). On gross examinations of the *Tgfbr1*^*iko*^*.Tgfbr2*^*iko*^ aortas, small intramural hematoma and tears were noted, but contained ruptures were not detected. The incidence of aortic pathology in the *Tgfbr1*^*iko*^*.Tgfbr2*^*iko*^ group was 47%, which was significantly lower than that (100%) in the *Tgfbr1*^*iko*^ group (Fig. 4C). In contrast to the severe aneurysmal degeneration of *Tgfbr1*^*iko*^ ATAs (Figs 1 and 2 and Supplementary Figures 3 and 4), *Tgfbr1*^*iko*^*.Tgfbr2*^*iko*^ ATA segments displayed isolated intimal/medial tears (5/17,29%) and small areas of intramural hematoma (3/17,18%), with medial thinning/depletion being less frequently detected (1/17,6%). Adventitial fibrosis was observed only in the ATAs with evident gross pathology. Overall, *Tgfbr1*^*iko*^*.Tgfbr2*^*iko*^ aortas displayed significantly less wall degeneration than *Tgfbr1*^*iko*^ aortas (Fig. 4D). Therefore, abrogation of the *Tgfbr2* signal ameliorated the aortic pathology induced by *Tgfbr1*^*iko*^. These *in vivo* observations indicate that SMC-specific *Tgfbr2,* in the absence of *Tgfbr1*, is deleterious to aortic wall homeostasis.

### Additional deletion of SMC-*Tgfbr2* inhibits activation of the ERK pathway in *Tgfbr1^iko^* aortas prior to aneurysmal degeneration

**Figure 5:**
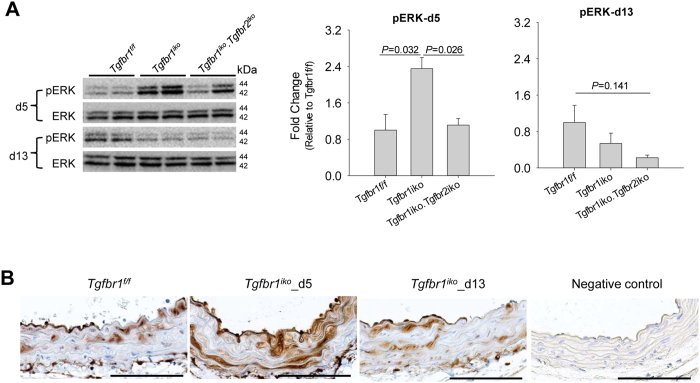
*Tgfbr1*^*iko*^ exaggerates extracellular-regulated kinase (ERK) signaling in the aortic wall prior to causing structural degeneration. (**A**) Production of pERK in *Tgfbr1*^*f/f*^, *Tgfbr1*^*iko*^, and *Tgfbr1*^*iko*^*.Tgfbr2*^*iko*^ ATA segments (*n* = 5 for each group). Intensity of pERK was normalized to that of ERK and then calibrated to that of *Tgfbr1*^*f/f*^ ATA segments. Data were analyzed using one-way ANOVA. (**B**) Immunohistochemistry assays for localization of pERK in *Tgfbr1*^*iko*^ and *Tgfbr1*^*f/f*^ ATA segments. Specimens stained with isotype-matched IgGs served as a negative control. Note the intense staining of pERK in cells located in the medial layer of *Tgfbr1*^*iko*^ ATAs. The panel at the end of the row represents the negative control where the primary antibody (Ab) was replaced with the blocking buffer. Scale bars: 100 μm.

Activation of the ERK pathway has been identified as a major molecular event for aortic aneurysm development in various animal models^7^,^12^,^29^. To explore the role of the ERK pathway in *Tgfbr1*^*iko*^ aortas, we evaluated the production and phosphorylation of pERK1/2 at various time points with western blotting assays. Compared with *Tgfbr1*^*f/f*^ controls, *Tgfbr1*^*iko*^ aortas produced two times more pERK1/2 by the time of completion of tamoxifen injection (d5, *n* = 5 per genotype), and this early augmentation was blunted by *Tgfbr2*^*iko*^. However, at the time that aortic rupture frequently occurred (d13, *n* = 5 per genotype), the production of pERK1/2 in *Tgfbr1*^*iko*^. *Tgfbr2*^*iko*^ aortas returned to baseline levels (Fig. 5A), and immunohistochemistry (IHC) assays revealed a trend of reduction of pERK1/2 in cells located in the media of *Tgfbr1*^*iko*^ ATA segments (Fig. 5B). These assays uncovered a genotype-specific correlation between the exaggeration of early ERK signaling and subsequent development of aortic pathology, suggesting a role for the ERK pathway in *Tgfbr1*^*iko*^-driven aortic disease.

An unsolved puzzle in the study of TGFβ signaling disorders is the “TGFβ paradox”, which is characterized by increased pSMAD2 abundance in cells with defective TGFβ signaling components^7^,^30^. We measured pSMAD2 in our models, and not surprisingly, ATA segments deficient in *Tgfbr1*, *Tgfbr2*, or both exhibited impaired rather than enhanced SMAD2 phosphorylation (Supplementary Figure 7A, *n* = 5 per genotype), and IHC assays revealed that the reduction in pSMAD2 production was attributed primarily to cells located in the tunica media of ATA segments (Supplementary Figure 7B). In the absence of *Tgfbr1*, *Tgfbr2* may assemble a signaling complex with other type I receptors, such as Alk1, to propagate the TGFβ signal through SMAD1/5/8, and this promiscuous receptor-receptor interaction may be an important mechanism of tissue fibrosis^28^. Therefore, we evaluated the production of pSMAD1/5/8 in our model (*n* = 5 per genotype). As the disease progressed to d13, the level of pSMAD1/5/8 became significantly higher in *Tgfbr1*^*iko*^ than in *Tgfbr1*^*f/f*^ ATA segments. However, pSMAD1/5/8 was also augmented in *Tgfbr2*^*iko*^ and *Tgfbr1*^*iko*^.*Tgfbr2*^*iko*^ aortas, with no differences among genotypes (Supplementary Figure 8). Thus, SMAD-dependent pathways may not be critical to the development of *Tgfbr1*^*iko*^-driven aortic disease.

### Pharmaceutical inhibition of ERK phosphorylation prevents aneurysmal degeneration of *Tgfbr1^iko^* aortas

**Figure 6:**
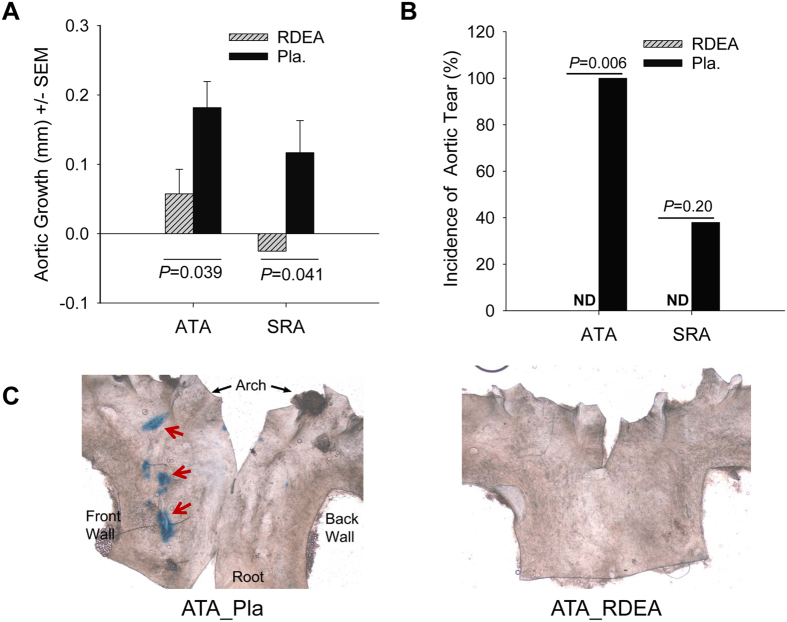
Inhibition of ERK phosphorylation effectively protects *Tgfbr1*^*iko*^ aortas from aneurysmal degeneration. (**A**) Changes in the diameter of RDEA-119-treated (RDEA, *n* = 7) or placebo-treated (Pla, *n* = 8) *Tgfbr1*^*iko*^ aortas in 14 days. Data were analyzed using the unpaired *t*-test. (**B**) Incidence of intimal/medial tears in *Tgfbr1*^*iko*^ aortas that were treated with RDEA-119 or placebo. Data were analyzed using Fisher’s exact tests. ND = not detected. (**C**) *En face* microscopy of the luminal surface of RDEA-119- or placebo-treated ATA segments. Focal intimal/medial tears were stained blue (indicated by red arrows), due to extravasation of Evans Blue, which was injected into mice prior to sample collection.

The tight genotype-specific correlation between the early upregulation of pERK1/2 and the subsequent development of aortic disease raises the question of whether this correlation reflects causation. A previous study indicated that RDEA-119, a small chemical compound, selectively inhibits ERK phosphorylation and attenuates aortic dilation in MFS mice^31^. Therefore, we treated *Tgfbr1*^*iko*^ mice with RDEA-119 (*n* = 7) and compared them to the solvent-treated controls (*n* = 8). Consistent with that study, RDEA-119 significantly inhibited ERK phosphorylation in the aortic wall (Supplementary Figure 9) and attenuated dilation of *Tgfbr1*^*iko*^ aortas (Fig. 6A). Although the mice were treated with the same dosage as previously reported^31^, we noticed that animals that were treated but not in the control group began to lose weight a week after RDEA-119 treatment. Thus, we had to stop the experiment prematurely after 2 weeks. Because the aortic pathology in *Tgfbr1*^*iko*^ aortas at this early time point was dominated by scattered intimal/medial tears (Supplementary Figure 4), we decided to use Evans Blue staining to catch aortic tears that would have otherwise been missed during routine histological evaluations. Aortic ruptures were not noted in either group. Extravasation of Evans Blue was not detected in any aortas of RDEA-119-treated mice but was present in all ATA segments and three of eight SRA segments in placebo-treated mice (Fig. 6B), indicating that treatment with RDEA-119 fully prevents intimal-medial tears at the early stage. Representative images showing areas of extravasation of Evans Blue (indicated by arrows) are shown in Fig. 6C.

### Losartan rescues the *Tgfbr1^iko^* phenotype without interrupting ERK phosphorylation

**Figure 7:**
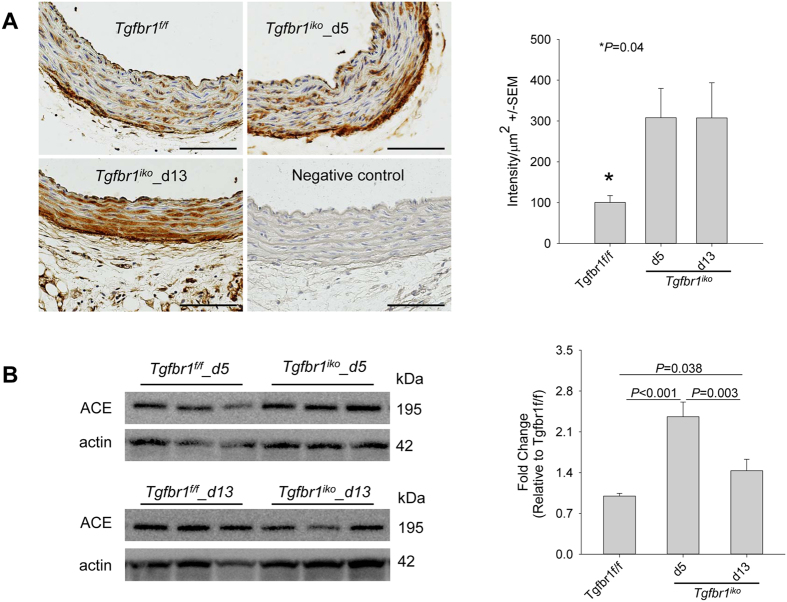
Angiotensin-converting enzyme (ACE) is upregulated in *Tgfbr1*^*iko*^ aortas prior to discernable aneurysmal degeneration. (**A**) Immunohistochemistry assays for ACE production in ATAs at the indicated time points (*n* = 5 per group). Specimens stained with isotype-matched IgGs served as a negative control. Note the intense staining of ACE in cells of the tunica media. (**B**) Western blotting assays for ACE production in ATAs at the indicated time points (*n* = 5 per group). Data were analyzed using one-way ANOVA. Scale bar: 100 μm.

**Figure 8:**
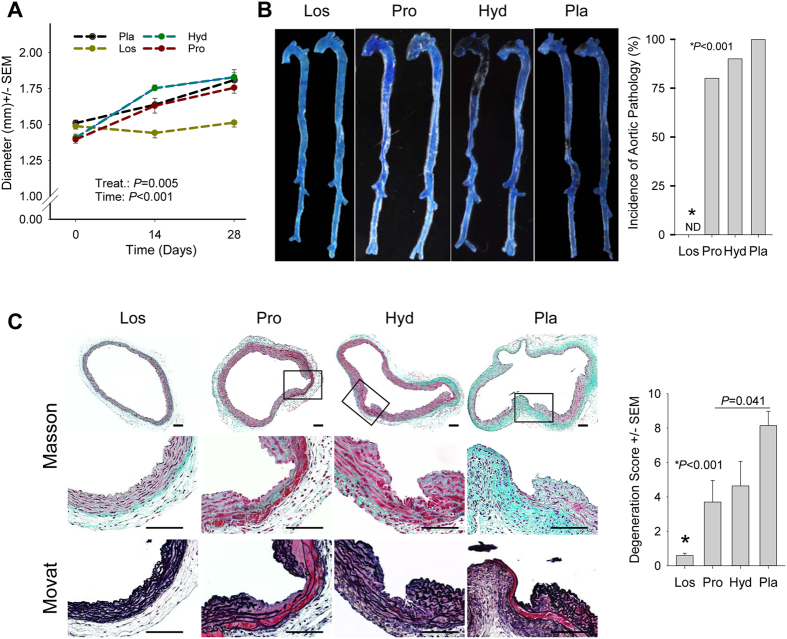
Losartan fully rescues the aortic phenotype of *Tgfbr1*^*iko*^. (**A**) Aortic dilation of the ATA segment of *Tgfbr1*^*iko*^ aortas that were treated with placebo (*n* = 9), hydralazine (Hyd, *n* = 12), propranolol (Pro, *n* = 10), or losartan (Los, *n* = 10). Data were analyzed using two-way repeated measurement ANOVA. (**B**) Gross evaluation of *Tgfbr1*^*iko*^ aortas that were treated with the indicated drugs. Data were analyzed with one-way ANOVA. (**C**) Histological evaluation of *Tgfbr1*^*iko*^ ATA segments that were treated with the indicated drugs. Note the improvement in the histology of the ATA after losartan treatment. Data were analyzed with one-way ANOVA.

A large body of evidence suggests an important role for the AngII/AT1R signaling pathway in the pathogenesis of thoracic and abdominal aortic aneurysms^1^,^32^. In addition, an interaction between TGFβ and AngII/AT1R signaling pathways was recently demonstrated in MFS and LDS mice^12^,^33^, thus calling for further mechanistic elucidation^32^. To this end, we evaluated the role of the AngII/AT1R axis in the aneurysmal degeneration of *Tgfbr1*^*iko*^ aortas. Compared with *Tgfbr1*^*f/f*^ controls, *Tgfbr1*^*iko*^ induced a three-fold increase of angiotensin-converting enzyme (ACE) in the tunica media of the aortic wall, and this elevation persisted at d13 (Fig. 7A). ACE production was further quantified with western blotting assays. Consistent with the observation of IHC assays, an upregulation of ACE production was significant at both d5 and d13. However, a reduction in ACE levels was observed at d13 compared with d5 (Fig. 7B). Because the upregulation of ACE was located primarily in medial SMCs (Fig. 7A), the reduction was most likely a result of the occurrence of intimal/medial tears and intramural hematoma that altered the cellular and protein compositions of the aortic tissue at this time point (Supplementary Figure 4C). To determine the importance of the AngII/AT1R axis in *Tgfbr1*^*iko*^-driven aortic disease, we disrupted this axis in *Tgfbr1*^*iko*^ mice with pharmaceutical intervention. The effects of the treatment were then evaluated over a 28-day period. Mice in the experimental group were treated with losartan (*n* = 10), a specific AT1R inhibitor, and mice in the control groups were treated with hydralazine (*n* = 12), propranolol (*n* = 10), or placebo (*n* = 9) to assess the effect of lowering hemodynamic stress to the aortic wall. As expected, losartan, hydralazine, and propranolol lowered systolic blood pressure by 22–28 points, with no statistically significant differences among treatments (Supplementary Figure 10). Ultrasound scanning of aortas at various time points showed that losartan significantly inhibited aortic dilation. This therapeutic effect was not a result of reduction in blood pressure, as animals treated with hydralazine or propranolol exhibited aortic dilation equivalent to the placebo controls (Fig. 8A). Additionally, losartan treatment normalized aortic structure, resulting in a morphology that was indistinguishable from that of *Tgfbr1*^*f/f*^ controls when evaluated with gross examination (Fig. 8B). Evidence of medial thinning, intramural hematoma, intimal/medial tears, or contained rupture was not detected in losartan-treated aortas (Fig. 8C). In contrast, lowering blood pressure alone failed to normalize aortic histology (Fig. 8B,C). Interestingly, propranolol seemed to attenuate aneurysmal degeneration, as compared with placebo (Fig. 8C). This improvement may be attributable to the drug effect of propranolol on heart rate and blood pressure, both of which lead to a significant reduction in wall stress. Additionally, aortic rupture did not occur when blood pressure was controlled at a relatively low level. This is in comparison to placebo-treated group, in which 2 out of 9 mice died from aortic rupture, indicating an impaired mechanical strength of *Tgfbr1*^*iko*^ aortas. One of the mechanisms proposed for the therapeutic effect of losartan on the development of aortic aneurysms is the inhibition of ERK phosphorylation^31^,^34^. As our results show that both RDEA-119 and losartan ameliorated aortic pathology in *Tgfbr1*^*iko*^ animals, we evaluated whether the therapeutic effect of losartan was achieved via inhibiting ERK phosphorylation. *Tgfbr1*^*iko*^ mice were treated with losartan or placebo and specimens were collected at d5 (*n* = 5 for each group) and d13 (*n* = 5 for each group) to examine ERK phosphorylation. Surprisingly, losartan-treated aortas produced similar amounts of pERK1/2 as placebo-treated aortas at various time points (Supplementary Figure 11), suggesting that ERK phosphorylation was not inhibited by losartan in *Tgfbr1*^*iko*^ aortas.

## Discussion

TGFβ is indispensable for vasculogenesis in embryos^35^. Studies focusing on specific cell lineages have also demonstrated the importance of SMC-specific TGFβ in vascular development particularly patterning of aortas^21^,^22^,^36^. In the postnatal life, however, TGFβ is frequently considered as a culprit for vascular diseases, such as hypertension, atherosclerosis, and stenosis/restenosis^20^. Despite the recognition of its role in governing tissue homeostasis^28^, its importance in maintaining the structural integrity of aortic walls was not appreciated up until recent studies showing that conditional deletion of SMC-specific *Tgfbr2* in mice at an age of 6 weeks results in aortic aneurysm formation and dissection^26^,^27^. A subsequent study showed that neutralization of pan-specific TGFβ isoforms at the early postnatal stage (<P45) increases the incidence of aortic rupture in MFS mice^37^, suggesting a role for TGFβ in aortic wall maturation. Consistent with these reports, the present study demonstrates that deletion of SMC-specific *Tgfbr1*, the physiologic partner of *Tgfbr2,* also caused the collapse of aortic structure, which was manifested by aortic rupture, intimal-medial tears, intramural hematoma, and medial thinning/depletion.

Although ligand-ligand and ligand-receptor interactions are highly promiscuous among TGFβ superfamily members, it is believed that *Tgfbr1* forms receptor complexes primarily with *Tgfbr2* to transduce the TGFβ signal in SMCs^20^. Theoretically, disruptions in *Tgfbr1* or *Tgfbr2* would lead to the same phenotype. In agreement with this prediction, the phenotype of TGFBR1 mutations is generally indistinguishable from that of TGFBR2 mutations in patients and animal models^1^,^12^, though various sites of mutation in each gene may account for its wide spectrum of clinical presentations^38^. Our experiments, however, identified different phenotypes for *Tgfbr1*^*iko*^ and *Tgfbr2*^*iko*^ aortas, with *Tgfbr1*^*iko*^ causing more severe aortic pathology than *Tgfbr2*^*iko*^ in adult mice. One explanation for this observation is that *Tgfbr1* signaling governs a wider spectrum of biological processes than *Tgfbr2* signaling in SMCs, as *Tgfbr1* may assemble receptor complexes with type II receptors in addition to *Tgfbr2*^39^. However, this plausible speculation could not explain the finding that *Tgfbr1*^*iko*^-induced pathology was significantly ameliorated by additional *Tgfbr2*^*iko*^. An alternative explanation is that *Tgfbr2* signaling remains activated in the absence of *Tgfbr1* and that this *Tgfbr2*-dependent signal contributes to the aortic pathology observed in *Tgfbr1*^*iko*^ mice. Although *Tgfbr2* is capable of self-phosphorylation, it requires a type I receptor to trigger the intracellular signaling cascade^28^. In cells lacking both *Alk1* and *Tgfbr1*, TGFβ is unable to alter the profile of global gene expression^40^. Together with our results, these findings raise the hypothesis that the availability of *Tgfbr2* in the absence of *Tgfbr1* triggers the promiscuous binding of *Tgfbr2* to Alk1, which leads to an aberrant TGFβ signal in SMCs that subsequently causes aneurysmal degeneration of the aortic wall. Li *et al*. reported that the impact of *Tgfbr2*^*iko*^ is age-dependent, with its deleterious effects being more severe in immature than in mature aortas^26^. We confirmed this observation in our study (data not shown). However, our data also showed that, in contrast to *Tgfbr2*^*iko*^, *Tgfbr1*^*iko*^ remains to be devastating to mature aortas. Although the underlying mechanisms have yet to be defined, the diminished age-dependent protective effect under conditions of *Tgfbr1*^*iko*^ may be attributable to the abnormal *Tgfbr2* signal.

Aortic aneurysms resulting from the mutation of genes that are directly or indirectly committed to TGFβ signaling display enhanced activation of both SMAD-dependent and -independent pathways, as evidenced by the nuclear accumulation of pSMAD2 and pERK^1^,^31^. Further mechanistic studies with MFS mice demonstrate that an inhibition of ERK activation suppressed aortic dilation and improved aortic wall structure^31^. The neutralization of pan TGFβ ligands inhibited ERK phosphorylation and attenuated aneurysmal degeneration of the aortic wall^37^. These findings indicate that the intensified ERK signaling acts as a driving force for the aortic phenotype of the MFS. Our data show that genotype-specific alterations in pERK1/2 production during the early stages correlated with the subsequent phenotypic severity. Furthermore, inhibition of ERK phosphorylation prevented aortic dilation and the occurrence of intimal/medial tears in *Tgfbr1*^*iko*^ aortas. These results reaffirm the theory that excessive ERK activity is deleterious to aortic wall homeostasis. We also showed that the early elevation of ERK phosphorylation was limited to a narrow window prior to the occurrence of intimal/medial tears, indicating that an early ERK elevation sets the stage for subsequent aneurysmal degeneration to occur. This finding is consistent with the observations that the contribution of β-arrestin2 and miR29b to aortic dilation is limited to the early stage in MFS mice^41^,^42^. Pathological exaggeration of the ERK signal can lead to activation of several pathways including enhanced production of MMP2 and MMP9^43^. Further studies to characterize the relationship between the temporal changes of ERK phosphorylation and activation of the downstream pro-aneurysmal molecular cascades will provide a more in-depth understanding of ERK’s role in regulating the phenotypic expression of *Tgfbr1*^*iko*^ aortas.

Although changes in SMAD-dependent pathways, including pSMAD2 and pSMAD1/5/8, were observed in our study, a correlation between these changes and phenotypic expression was not detected, indicating a modest role for the canonical pathways in *Tgfbr1*^*iko*^-driven aortic disease. This finding is consistent with the report that an upregulation of pSMAD2 in the aortic tissue was not evident prior to severe structural degeneration in LDS mice^12^. In the established human aortic aneurysms, an increased abundance of nuclear pSMAD2 has been repeatedly documented^1^,^15^. Further investigations have unraveled the epigenetic upregulation of the total SMAD2 production as a major contributor to the enhanced pSMAD2 accumulation in SMCs^15^. It is intriguing that the SMC-biased pSMAD2 confers a protective effect against aortic dissection by suppressing proteolytic activity^15^,^16^. This notion is further supported by experimental studies. For example, studies from us^24^, as well as other groups^26^,^27^, have demonstrated that a disruption of the basal levels of TGFβ activity breaks the aortic wall homeostasis, leading to aortic aneurysm formations and dissections. Serological removal of pan TGFβ isoforms exacerbates the incidence of aortic rupture in mice receiving chronic infusion of angiotensin (Ang) II^23^,^44^. In the current study, we observed a reduced SMAD2 phosphorylation across various genotypes. While the blunted SMAD2 signal is not a primary contributor to the differential phenotypic expression, it might have created a context that renders the aortic wall more susceptible to the complex dysregulation of TGFβ signaling, such as the abnormal TGFBRII signal induced by*Tgfbr1*^*iko*^. Therefore, our results underscore the importance of both basal TGFβ activity and the control of ERK activation in maintaining the aortic wall homeostasis.

Despite the limited benefits of losartan in Marfan patients^45^, experimental and clinical studies have consistently demonstrated an important role for the AngII/AT1R axis in the development of aortic aneurysms^12^,^33^,^46^. We also show that losartan treatment fully rescued the *Tgfbr1*^*iko*^ aortic phenotype. Studies from other groups suggest that the therapeutic effects of losartan are achieved via inhibition of the ERK pathway in the aortic wall^31^,^37^. However, we found that losartan treatment had only a modest impact on the production of pERK in *Tgfbr1*^*iko*^ aortas, indicating that the deleterious effects of the AT1R signaling may not be processed via stimulating ERK-phosphorylation in this particular context. An alternative explanation for this observation is that the therapeutic effect of RDEA-119 in *Tgfbr1*^*iko*^ mice might be achieved through an off-target effect. Although we could not exclude this possibility, this compound is highly selective to MEK^47^, and its cardiovascular toxicity has been found to be modest^48^. This suggests that inhibition of ERK phosphorylation accounts for the treatment effect of RDEA-119 in our experiments. In addition to ERK, the AT1R signal can activate several other kinases such as PKC, JNK, Akt, and JAK, all known to play a critical role in promoting aneurysmal degeneration^26^,^31^,^49^,^50^. Oxidative stress, another pro-aneurysmal mediator, can also be promoted by the AT1R signal via mechanisms independent of ERK activation. In SMCs, for instance, AT1R signal stimulates the production of reactive oxygen species (ROS) via ARF6-mediated Nox1 upregulation^51^. It appears that the therapeutic benefits of AT1R blockade can be achieved via a disruption of multiple pro-aneurysmal pathways, with the primary target varying among animal models. A recent study showed that hydralazine inhibits PKCβ-mediated ERK phosphorylation and prevents aortic dilation of MFS mice^52^. We included hydralazine as a control to assess losartan’s effect of lowering blood pressure in this study and did not detect any protective effects for this drug, indicating a modest role for PKCβ in mediating aneurysmal degeneration of *Tgfbr1*^*iko*^ aortas.

The mechanisms responsible for the activation of the local AT1R during aortic aneurysm development remain unclear. Our data show that the production of ACE was upregulated in *Tgfbr1*^*iko*^ aortas, particularly in the medial layer, indicating AngII-mediated AT1R activation. This mechanism appears to not be unique to the *Tgfbr1*^*iko*^, as the *Myh11* mutation^53^ and fibulin4 deletion^54^ are both associated with increased ACE production in the aortic wall. In addition to its ligand-dependent function, AT1R may act as a mechanosensor and can be activated in the heart^55^ and myogenic arteries^56^ in a ligand-independent fashion. Future studies are warranted to determine the importance of this mechanism in aortic aneurysm formation^57^.

Our study has a few limitations. First, mouse models were created via a postnatal homozygous gene deletion rather than a germline heterozygous gene mutation, as observed in TAAD and LDS patients^1^. Therefore, these models cannot be considered as models of TAAD or LDS. However, the *Tgfbr1*^*iko*^ model did recapitulate several key pathologies observed in TAAD and LDS patients, including aortic rupture, intimal-medial tears and dissections, and progressive aortic dilation^29^. It offers a unique platform for investigating the timing of TGFβ function over the course of aortic wall maturation, thus improving our understanding of TGFβ signaling disorders in the context of aortic aneurysm formation. In addition, although we linked *Tgfbr1*^*iko*^ to the activation of ERK and AngII/AT1R signaling pathways in *Tgfbr1*^*iko*^ aortas, it remains unclear whether this activation occurs via cell-autonomous mechanisms or is secondary to intermediate responses, such as mechanosensing at the tissue level^57^. Studies from other groups have shown that the local renin-angiotensin system (RAS) can be activated as a result of an upregulation of AT1R^34^ and ACE^54^ in aneurysmal aortas. While our study focused on ACE production, it is possible that the expression of AT1R is also increased in *Tgfbr1*^*iko*^ aortas. Finally, the ligand and the receptor complex for the aberrant TGFBRII signaling still remain to be determined. Further studies to quantify TGFBRII levels will help to understand the mechanism by which the loss of TGBRI triggers the aberrant TGFBRII signal in *Tgfbr1*^*iko*^ aortas. Our examination of a few selected TGFβ responsive mediators involved in canonical and non-canonical TGFβ signaling pathways was unable to identify any measurable readouts in SMCs lacking TGFBRI or TGFBRII after treatment with TGFβ1 (Supplementary Figure 2). However, we would not interpret this observation as evidence that excludes TGFBRII activity in *Tgfbr1*^*iko*^ SMCs because of the limitation of the *in vitro* SMC model to recapitulate the *in vivo* regulation of TGFβ signaling components. For example, SMCs with TGFβ2 mutations produced higher levels of TGFβ2 in the aortic tissue, but synthesized significantly less TGFβ2 in culture when compared with wild type controls^6^. Studies using high throughput approaches and the selected collection of the medial layer of the aortic tissue are warranted to screen for responsible mediators in order to shed light on proximal signaling components such as the ligand and the type I receptor members for the aberrant TGFBRII signal.

In summary, we have provided evidence suggesting that the *TGFBR1* signaling pathway in SMCs is critical to the maintenance of structural integrity and homeostasis of aortas at an adult age. Our results suggest a novel mechanism for TGFβ signaling disorders. Namely, *Tgfbr1*^*iko*^ triggered an abnormal *Tgfbr2* signal that is deleterious to the aortic wall. In addition, ERK and AngII/AT1R signaling pathways were also activated in the aortic wall following *Tgfbr1*^*iko*^. Activation of these pathways caused the rapid collapse of aortic homeostasis, thereby promoting aortic aneurysm formation and dissection (Supplementary Figure 12).

## Materials and Methods

### Mice

The *Tgfbr1*^*f/f* ^58 and *Tgfbr2*^*f/f* ^59 strains were kindly provided by Dr. Karlsson (Lund University) and Dr. Moses (Vanderbilt University), respectively. The *Myh11-CreER*^*T2*^ strain^60^ was obtained from Dr. Weiser-Evans (University of Colorado) with the permission of Dr. Offermanns (Semmelweis University). The *Gt(ROSA)26sor* reporter strain, colloquially referred to as “*R26R*”, was purchased from Jackson Laboratory. These floxed mouse lines were crossed with *Myh11-CreER*^*T2*^ mice to establish colonies of *R26R.Myh11-CreER*^*T2*^, *Tgfbr1*^*f/f*^*.Myh11-CreER*^*T2*^, *Tgfbr2*^*f/f*^*.Myh11-CreER*^*T2*^, and *Tgfbr1*^*f/f*^*.Tgfbr2*^*f/f*^*.Myh11-CreER*^*T2*^ mice. We used male mice at 9–13 weeks of age. Each strain was back-crossed to C57BL/6 females for at least five generations before breeding with other strains. Our breeding results show that the *Myh11-CreER*^*T2*^ allele could be inherited by both male and female littermates. Because the transgene was initially inserted into the Y chromosome, chromosome-translocation of the *Myh11-CreER*^*T2*^ allele must have occurred in our colony. Male mice carrying the *Tgfbr1*^*f/f*^ but not the *Myh11-CreER*^*T2*^ allele were saved from a colony that produced female carriers of the transgene and used as wild-type controls.

### Animal treatment

Tamoxifen was administered via intraperitoneal injection (2.5 mg/day) for 5 consecutive days. The day of the first tamoxifen injection was considered to be d0. RDEA-119 (Selleckchem, S1089) was dissolved in 10% 2-hydroxypropyl-beta cyclodextrin (Sigma, 332607) in phosphate-buffered saline and administered twice daily by oral gavage at a dose of 25 mg/kg body weight^31^. Dissolvent was given to mice in the control group with the same volume and dosing schedule. Losartan was administered through a customized diet (Harlan) containing 1 mg active drug per gram of food, which delivered an estimated dose of 3 mg daily to a mouse consuming 3 grams of food per day. Hydralazine and propranolol were delivered to mice through drinking water at concentrations of 250 mg/l^61^ and 500 mg/l^11^, respectively. Blood pressure of these animals was measured with a non-invasive tail-cuff method (CODA, Kent Scientific, Torrington, CT USA)^24^. All treatments were started 1 week prior to the first dose of tamoxifen, except for RDEA-119 treatment, which began only 3 days before the first tamoxifen dose.

### Gross examination

When a hemothorax and/or hematoma were noted during necropsy, the mouse was diagnosed with an aortic rupture. Otherwise, animals were evaluated for intimal/medial tears on d13 and advanced aortic wall remodeling on d28. These time points were chosen based on our pilot observation which showed complete penetrance of intimal/medial tears by d13 and grossly evident aortic pathologies by d28. For mice undergoing scheduled examination, 0.2 ml Evans Blue (5% in saline) was injected via tail vein 30 minutes prior to opening the chest. Immediately after opening the chest, 2.0 ml saline was injected into the left ventricle and drained from the right atrial appendage to remove blood and Evans Blue from the circulation. The use of Evans Blue to examine the changes of endothelial permeability in experimental aneurysms was invented several decades ago^62^. Other tracers such as horseradish peroxidase have also been used for the same purpose in previous studies^63^. We chose the use of Evans Blue staining to visualize the aortic tears because of its technical simplicity and high sensitivity to detect areas with a damaged endothelial barrier. More importantly, we have previously used scanning electron microscopy (SEM) to verify that areas with Evans Blue extravasation co-localize with one or more intimal/medial tears in our model^24^. Gross examination was then performed under an operating microscope by noting the presence and location of pathologies, including aneurysm formation, intramural hematoma, and contained rupture (Supplementary Figure 3). In addition to grossly evident pathologies, aortas with one or more spots of Evans Blue extravasation were also considered to be diseased. The incidence of aortic pathology for a given group was calculated as the percentage of mice with one or more of these aortic pathologies.

### Tissue collection

Each aorta was divided into four segments: ascending aorta (ATA); descending aorta (DTA), from the takeoff of the left subclavian artery to the diaphragm; suprarenal aorta (SRA), distal to the diaphragm and proximal to the renal arteries; and infrarenal aorta (AA). Aortic tissues were either snap-frozen in liquid nitrogen for mRNA and protein assays or perfusion-fixed with 10% neutral buffered formalin for histology. The aortic root was not collected and the distal end of the perfusion-fixed ATA segments were labeled with a suture to ensure the subsequent sectioning to begin at the proximal end. All assays in this study were performed with ATA specimens, due to the complete phenotypic penetrance of the disease in this segment.

### Histology and morphometry

Masson’s and Movat’s staining were performed as described previously^24^. ATA segments showing no grossly evident lesions but one or more spots of Evans Blue extravasation were opened longitudinally and evaluated *en face* to document early aortic lesions. X-gal staining (Mirus, MIR 2600) was performed on frozen sections (5.0-μm thickness), as per the manufacturer’s instructions. The medial area and length of internal and external elastic lamina were measured with ZEN lite (Zeiss) on cross sections and used for the approximation of medial thickness.

### Scoring system for quantifying the severity of aortic pathology

The severity of ATA pathology was quantified on a 5-point scale for intimal/medial tears, intramural hematoma, and medial thinning/depletion. Intramural hematoma was deemed when red blood cells and/or thrombi were found between elastic laminae. Medial thinning was considered when the thickness of the media was thinner than that of the surrounding area, and the absence of the tunica media was noted as “medial depletion”. Equal weight was assigned to each category, and an example is provided in Supplementary Figure 5. Two sections approximately 500 μm apart were scored for each specimen, and the average of the total scores was calculated to represent the degree of aneurysmal degeneration of that sample.

- Intimal/medial tears

- Multiple sites with one or more tears penetrating full wall thickness: 5
- Multiple sites with one or more tears penetrating >50% but <100% of wall thickness: 4
- Single tear penetrating >50% but <100% of wall thickness or multiple tears penetrating <50% of wall thickness: 3
- Single tear penetrating <50% of wall thickness: 2
- Elastic fiber breaks without tears: 1
- No tears or elastic fiber breaks: 0

- Intramural hematoma

- Diffused hematoma: 5
- Multiple hematomas involving multiple layers: 4
- Single hematoma involving multiple layers: 3
- Single hematoma restricted to a single layer but spreading >50% of the wall: 2
- Single hematoma restricted to a single layer and spreading <50% of the wall: 1
- No hematoma: 0

- Medial loss (thinning or depletion)

- Multiple sites of medial depletion: 5
- Loss >50% of media in >50% of the wall: 4
- Loss >50% of media in <50% of the wall: 3
- Loss <50% of media in >50% of the wall: 2
- Loss <50% of media in <50% of the wall: 1
- No medial loss: 0

### SEM

SEM was used to examine and characterize early aortic pathology, as previously described^24^.

### Ultrasound imaging

The development and progression of aortic pathology was monitored via ultrasound examination. A high-resolution Vevo 2100 Imaging System with a MS550D (25–55 MHz) linear array transducer was utilized (VisualSonics) to acquire images. The diameters of ATA and SRA segments were measured at the maximum point of systole, as previously described^24^.

### Immunohistochemistry assays

Assays were performed on formalin-fixed, paraffin-embedded sections. Citrate buffer- and heat-mediated antigen retrieval was carried out for pSMAD2 and pSMAD1/5/8 staining. Primary antibodies were purchased from Abcam (smooth muscle myosin heavy chain: ab53129), Santa Cruz (ACE: H-170), and Cell Signaling (pERK1/2: 4370; pSMAD2: 3101; and pSMAD1/5/8: 9511). Isotype-matched rabbit IgGs (Novus: NPB2-24893) served as a negative control. Antigen-specific signals were either detected with Alexa Fluor conjugated secondary antibodies (Life Technologies) or amplified with the ABC kit (Vector) and visualized via the DAB detection kit (Vector).

### Western blot

ATA segments were homogenized in 50 mM Tris-HCl buffer supplemented with phenylmethanesulfonyl fluoride (0.1 μM), leupeptin (10.0 nM), and a phosphatase inhibitor cocktail (Invitrogen). The concentration of the protein solution was determined with the BCA protein assay kit (Thermo Scientific, 23225). Total protein (5 μg) was separated with SDS-PAGE gels, and membranes were blotted with primary antibodies purchased from R&D (Tgfbr2, MAB532), Santa Cruz (Tgfbr1, SC398), and Cell Signaling (SMAD2, 5339; SMAD3, 9523; pSMAD3, 9520; ERK1/2, 4696, and others as detailed above). Immunoblots were developed with Lumigen substrate (#TMA-6) and imaged with x-ray films. The intensity of the band of interest was quantified with ImageJ software, and data were normalized to β-actin or total protein of the assayed molecules.

### Smooth muscle cell (SMC) culture and TGF-β1 treatment

Primary SMCs were explanted from *R26R;Myh11-CreER*^*T2*^, *Tgfbr1*^*iko*^, and *Tgfbr2*^*iko*^ aortas on the following day of the last dose of tamoxifen and cultured in DMEM/12 plus 10% fetal bovine serum, as described previously^64^. The outgrowth at passages 2–3 was examined with flow cytometry analysis to determine the percentage of cells producing α-actin. With our established protocol, we have been consistently separate murine aortic SMCs with purity greater than 95%. Cells at passages of 9–10 were stocked for experiments. During experiments, cells were seeded at a density of 1 × 10^5^ cells/cm^2^ and allowed to recover overnight. After a 24-hour serum deprivation, cells were treated with TGF-β1 (1.0 ng/ml, R&D, 240-B-002) for 1 or 24 hours and lysed with RIPA buffer supplemented with protease and phosphatase inhibitor cocktails (Invitrogen). Protein concentrations were measured with the BCA protein assay kit.

### Gene expression

The probe and primer sets were purchased from Applied Biosystems, and quantitative RT-PCR assays were performed following a previously described protocol^64^.

### Statistics

SigmaPlot version 13.0 was used for statistical analyses. Student *t*-tests, Fisher’s exact tests, Chi-square tests, one-way ANOVA, two-way repeated measurement ANOVA, and Kaplan Meier survival analyses were performed as appropriate, with *P* < 0.05 considered to be statistically significant. Datasets were evaluated using normality and equivalence variance testing. For those failing this evaluation, logarithmic and exponential transformations were employed to meet these requirements.

### Study approval

The Institutional Animal Care and Use Committee (IACUC) at the University of Florida approved this study and animal experiments were carried out in accordance with the approved guidelines.

## Additional Information

**How to cite this article**: Yang, P. *et al*. Smooth muscle cell-specific *Tgfbr1* deficiency promotes aortic aneurysm formation by stimulating multiple signaling events. *Sci. Rep.* **6**, 35444; doi: 10.1038/srep35444 (2016).

## Supplementary Material

Supplementary Information
