# Supplementary material for: Smooth muscle cell-specific Tgfbr1 deficiency promotes aortic aneurysm formation by stimulating multiple signaling events
Source: Sci Rep. 2016 Oct 14;6:35444. doi: 10.1038/srep35444 (PMC5064316; doi:10.1038/srep35444)
Supplement: Supplementary Information [file srep35444-s1.pdf]

Title: Smooth muscle cell-specific *Tgfbr1* deficiency promotes aortic aneurysm formation by stimulating multiple signaling events

Pu Yang[1, 3], Bradley M. Schmit[1], Chunhua Fu[1], Kenneth DeSart[1], S. Paul Oh[2], Scott A. Berceli[1, 4], Zhihua Jiang[1]

Supplementary Data

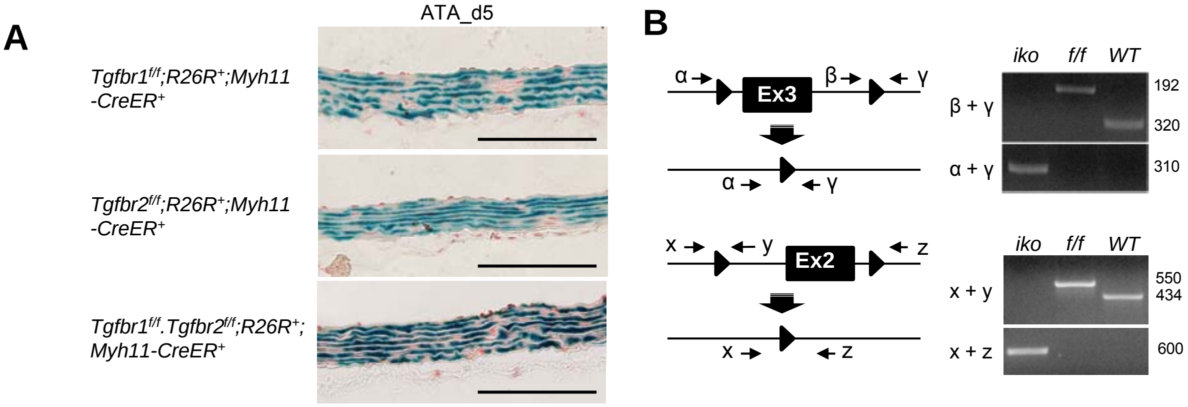

Supplementary Figure 1. The *Myh11*-driven Cre-loxP system facilitates efficient deletion of *Tgfbr1* and *Tgfbr2* in aortic SMCs. (**A**) X-gal staining. Note the robust recombination-events in the medial layer of the ATAs. Scale bars: 100 μm. (**B**) Genotyping results obtained with genomic DNA extracted from explanted SMCs and primers illustrated in the cartoon. Upper row, *Tgfbr1* deletion; Lower row, *Tgfbr2* deletion.

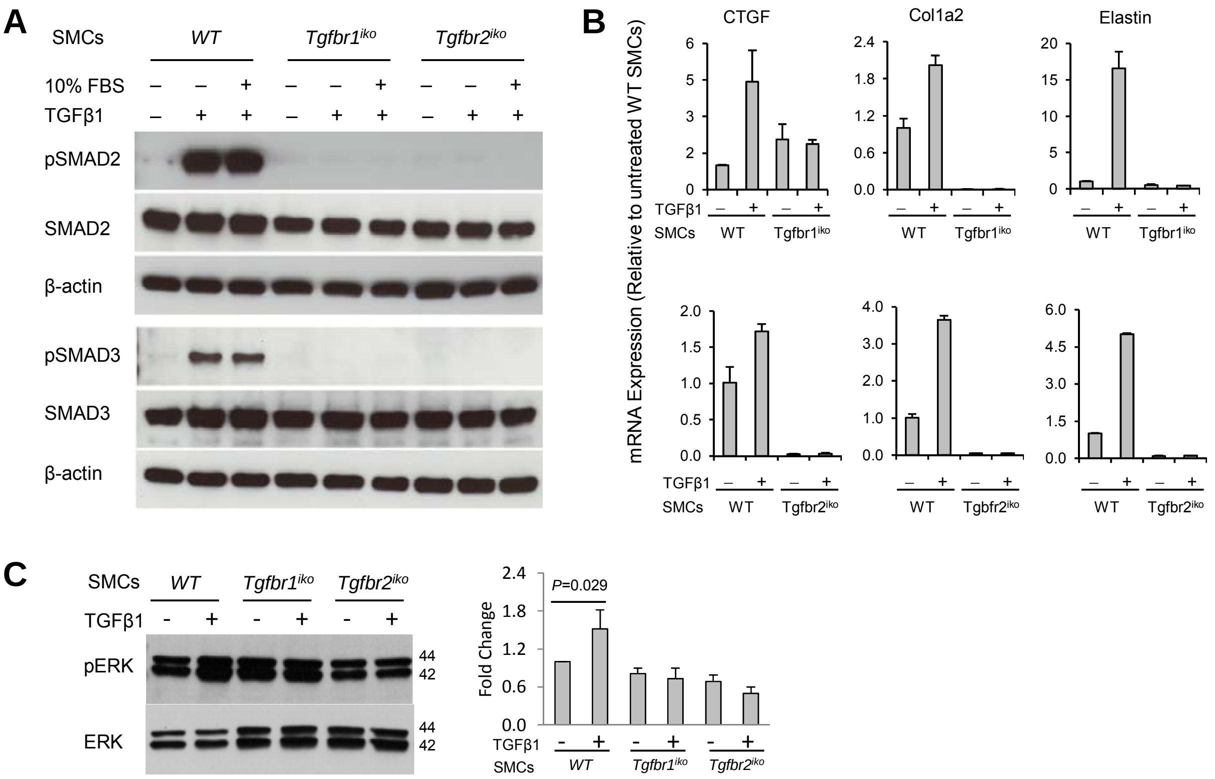

Supplementary Figure 2. *Tgfbr1*^{iko} or *Tgfbr2*^{iko} abrogates TGF-β-stimulated SMAD and ERK signaling in SMCs. (**A**) Western blotting assays for SMAD2 and SMAD3 phosphorylation. The addition of serum to TGF-β1 treatment was intended to create a more physiological context for TGF-β stimulation. (**B**) Expression of TGF-β responsive genes in SMCs with the indicated genotype. Levels of mRNA were determined with qRT-PCR. (**C**) Activation of ERK pathway in SMCs following TGF-β1 stimulation. Assays were performed in triplicates. SMCs were stimulated with TGF-β1 (1.0ng/ml) for one and twenty-four hours for protein and mRNA assays, respectively.

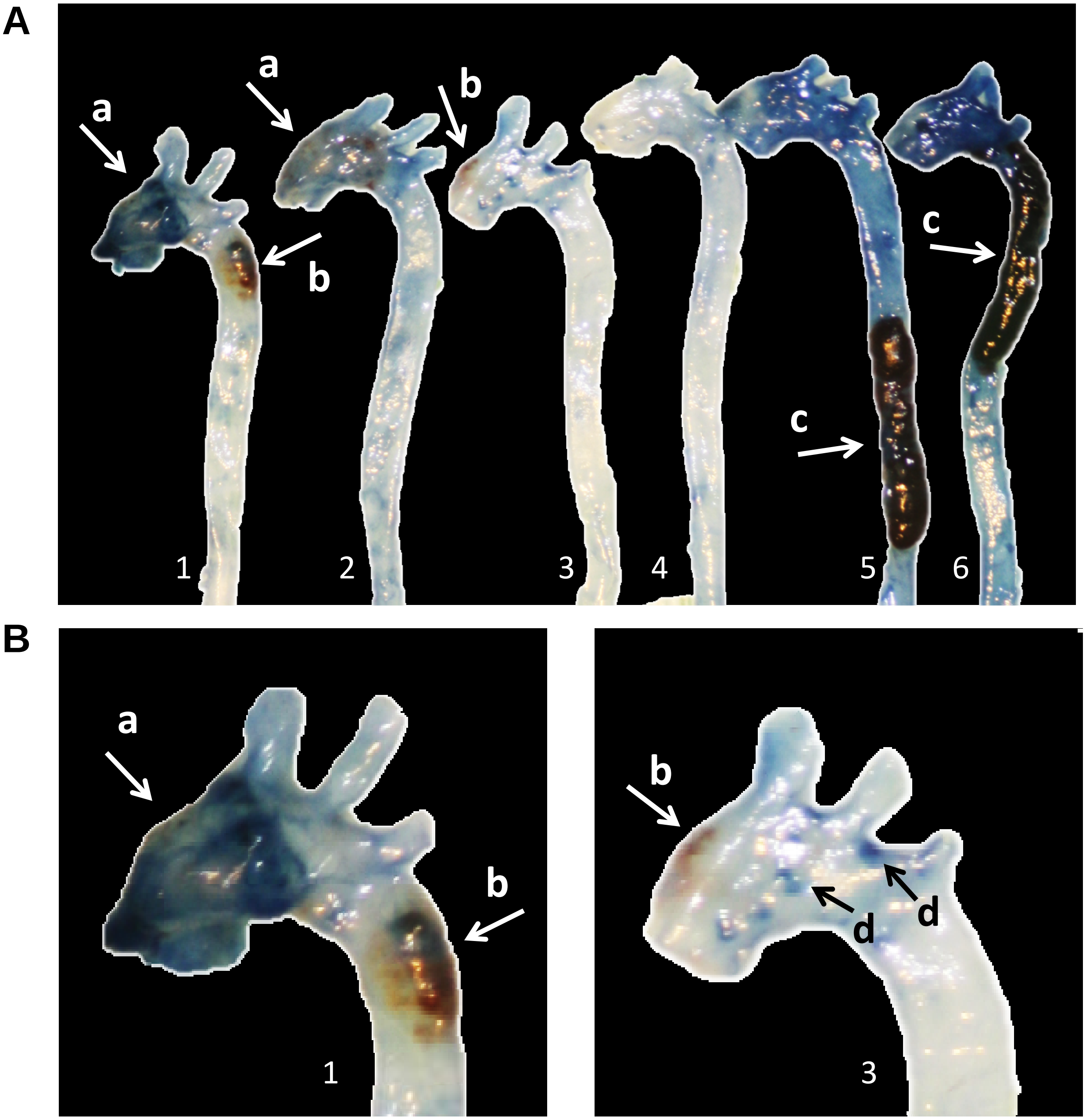

Supplementary Figure 3. Aortic pathologies detected on gross examination in *Tgfbr1*^{iko} aortas at d28. Evans blue (5% in saline) was injected through tail vein to mice 30 minutes before tissue collection. (**A**) Thoracic aortas. Arrows a, b, and c indicate the pathologies of aneurysmal dilation, intramural hematoma, and contained rupture, respectively. (**B**) Magnified view of the aorta #1 and #3. Arrow d points to areas with Evans blue extravasation through intimal/medial tears.

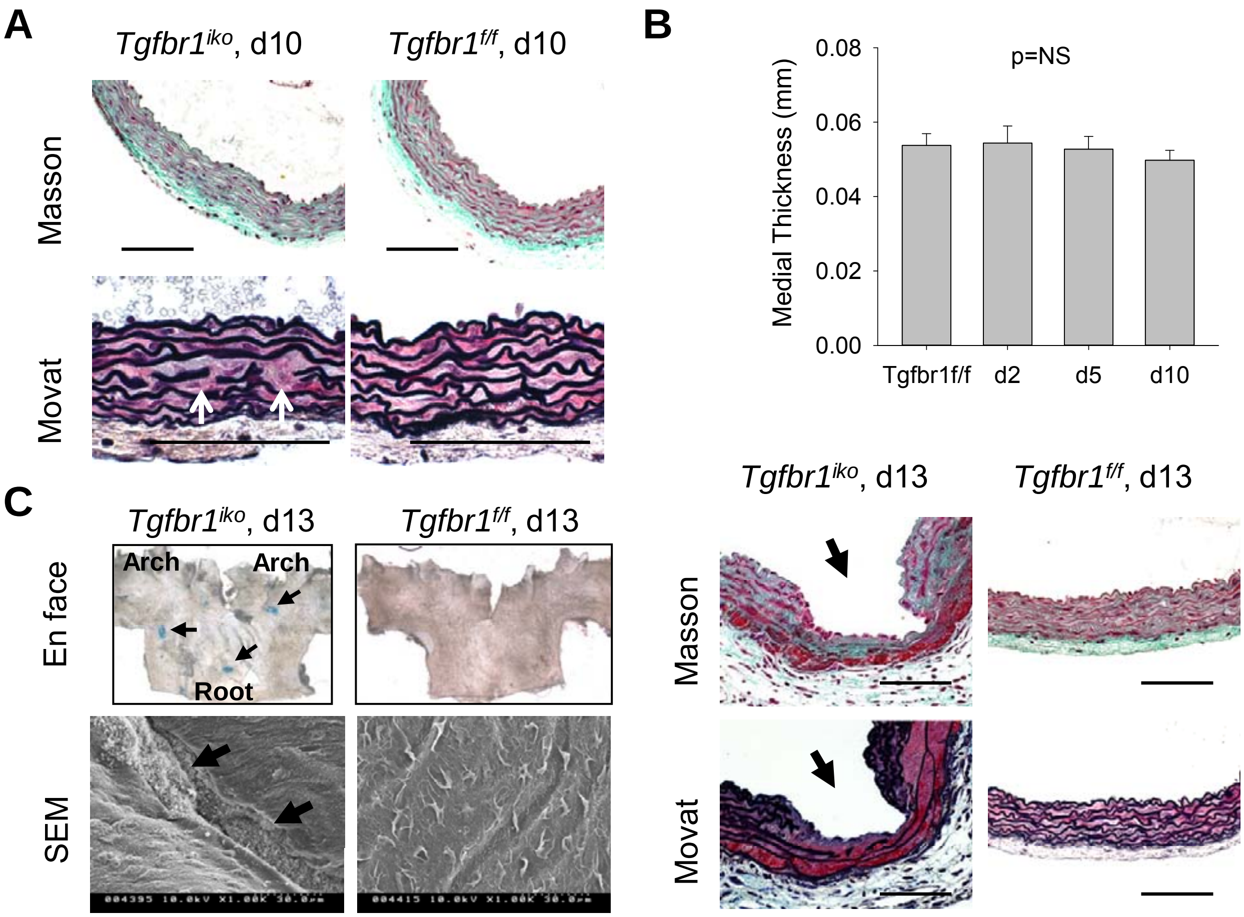

Supplementary Figure 4. Early pathology of *Tgfbr1*^{iko} aortas features isolated intimal/medial tears and intramural hematoma. (**A**) Histology of ATAs with indicated genotype on d10. Arrows point to elastic fiber breaks. (**B**) Medial thickness measured for *Tgfbr1*$^{f/f}$ (d10, *n*=7) and *Tgfbr1*^{iko} (*n*=6, 7, and 12 at d2, d5, and d10, respectively) ATAs. Data were analyzed using one-Way ANOVA. (**C**) Histology of ATAs with indicated genotype on d13. En face indicates luminal *en face* microscopy. Arrows point to areas with Evans blue extravasation. SEM denotes scanning electron microscopy. Masson's and Movat's staining images show areas of Evans blue extravasation correlates to intimal/medial tears (arrows). Scale bars: 100 μm.

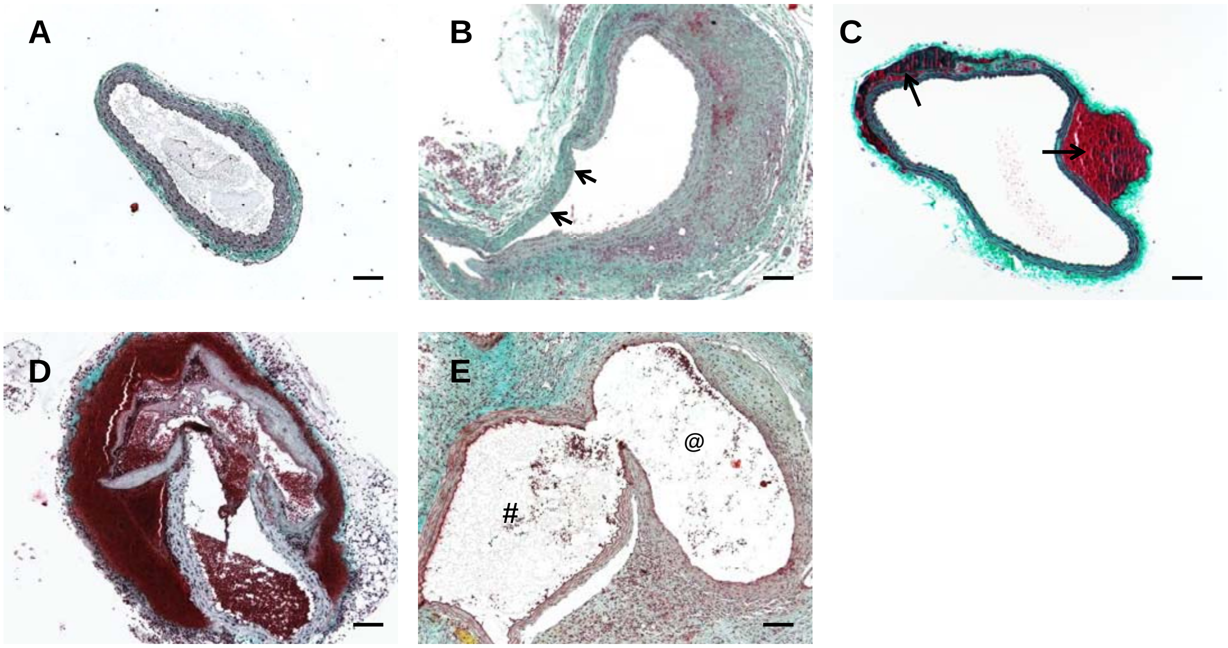

Supplementary Figure 5. Aortic aneurysms located in the SRA region displays similar pathological features as those located in the ATA region. Images represent Masson's trichrome staining of cross sections of the SRA specimens collected from different animals on d28. Panel A represents normal histology of *Tgfbr1*$^{f/f}$ SRA segments while panels B-E show medial depletion (B, arrows), intramural hematoma (C, arrows), rupture (D), and aortic dissection (E, #: true lumen; @: false lumen) detected in *Tgfbr1*^{iko} SRA segments, respectively. Scale bars: 100 μm.

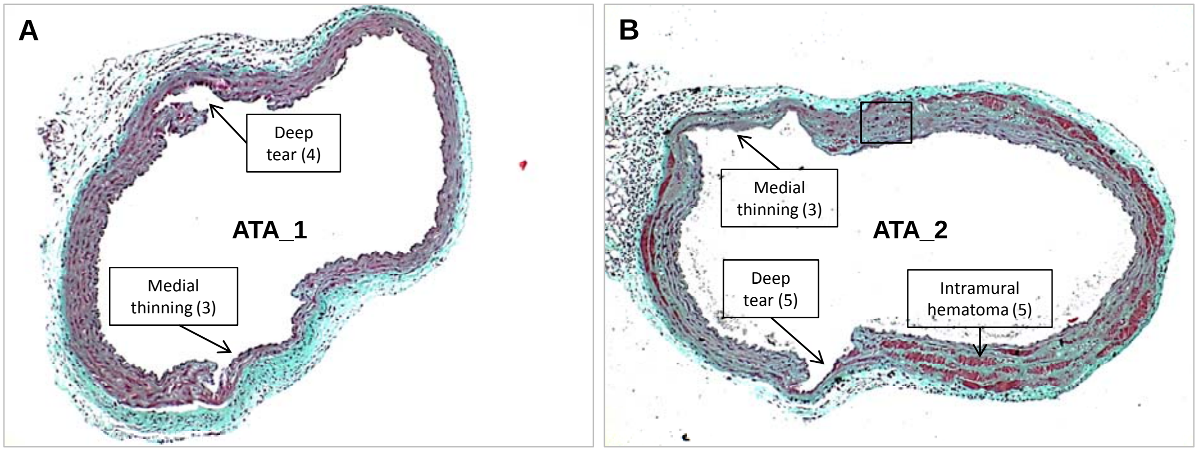

Sample	Intimal/ medial tears	Intramural hematoma	Medial thinning/ depletion	Total score
ATA_1	4	0	3	7
ATA_2	5	5	3	13

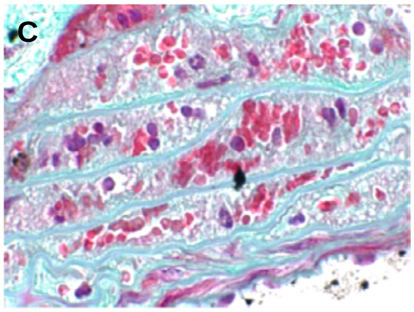

Supplementary Figure 6. Examples of using the proposed scoring system to quantify aortic wall degeneration. Masson's staining images (**A**) and (**B**) show ATA specimens collected from two individual *Tgfbr1*^{iko} animals. Pathologies present each specimen are specified in the text-boxes with a score for that pathology given in the parenthesis. Scores assigned to each specimen are summarized in the table and the total score is utilized to estimate the severity of aneurysmal degeneration. (**C**) Magnified view of the boxed area in B, showing presence of blood cells in space between elastic laminae.

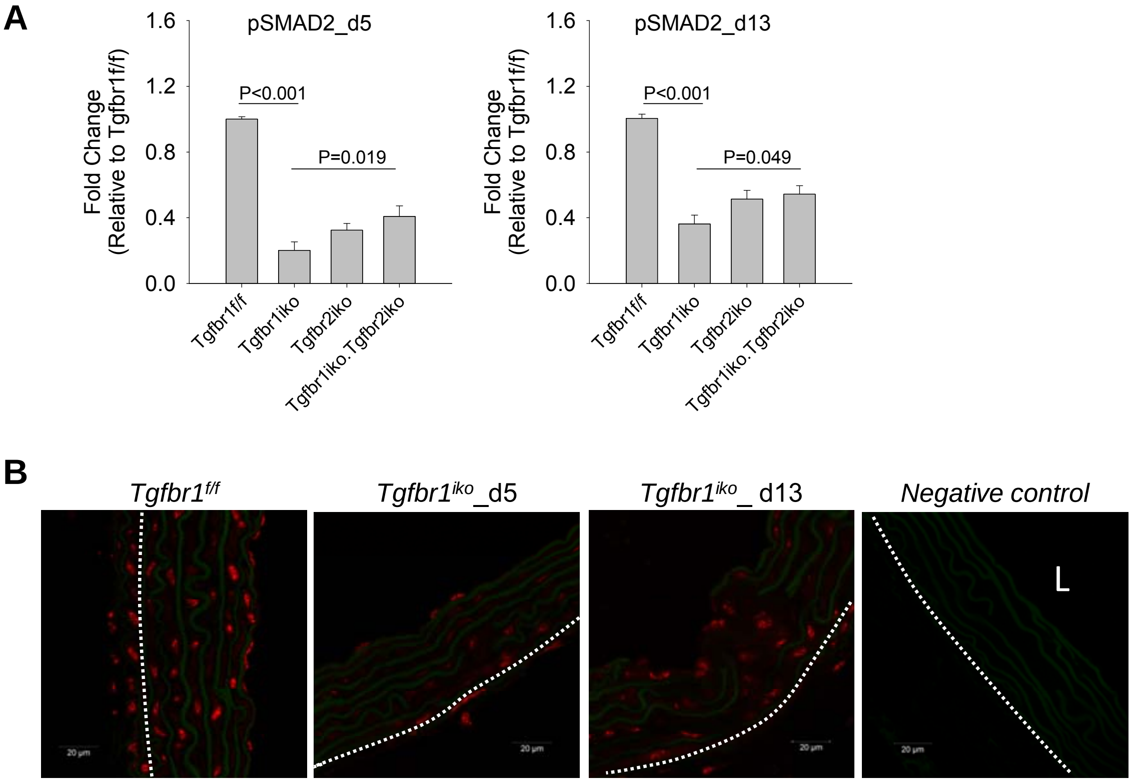

Supplementary Figure 7. Loss of SMC specific *Tgfbr1*, *Tgfbr2*, or both disrupts the SMAD2-mediated TGF-β signaling. (**A**) Quantification of the pSMAD2 immunoblots for *Tgfbr1*$^{f/f}$, *Tgfbr1*^{iko}, *Tgfbr2*^{iko}, and *Tgfbr1*^{iko}.*Tgfbr2*^{iko} ATAs (n=5 in each group) at the indicated time points. Statistics were obtained with one-way ANOVA. (**B**) Fluorescent IHC assays for pSMAD2 in ATAs. Red, pSMAD2; Green, auto-fluorescence of the elastic laminae. White dash lines indicate the medial-adventitial boarder. Specimens stained with isotype-matched IgGs served as a negative control. Note the reduced intensity of pSMAD2 staining in *Tgfbr1*^{iko} ATAs compared to the *Tgfbr1*$^{f/f}$ ATAs.

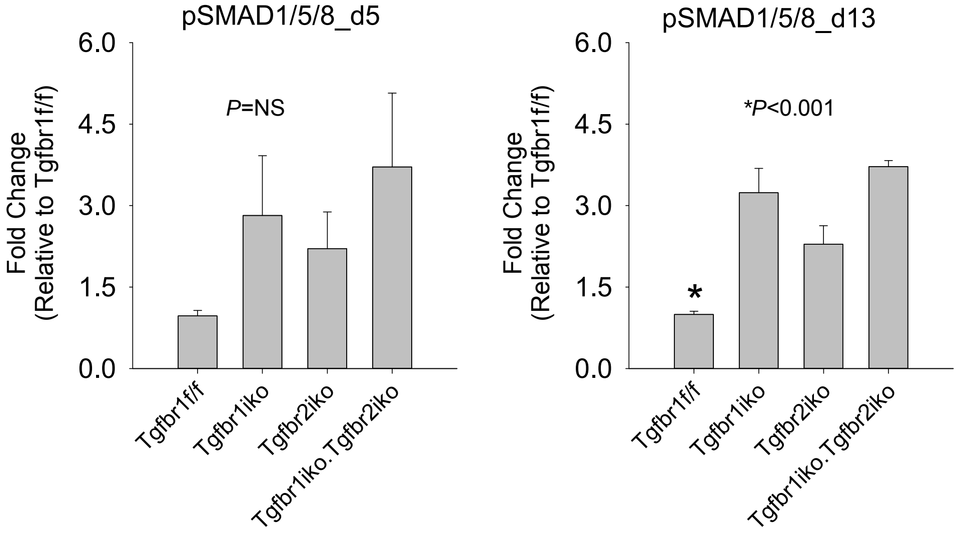

Supplementary Figure 8. Loss of SMC specific *Tgfbr1*, *Tgfbr2*, or both *Tgfbr1*and *Tgfbr2* increases the level of pSMAD1/5/8 in the aortic wall. Bar graphs show quantification of the pSMAD1/5/8 immunoblots produced by *Tgfbr1*$^{f/f}$, *Tgfbr1*^{iko}, *Tgfbr2*^{iko}, and *Tgfbr1*^{iko}.*Tgfbr2*^{iko} ATAs (*n*=5 in each group) at the indicated time points. Statistics were obtained with one-way ANOVA.

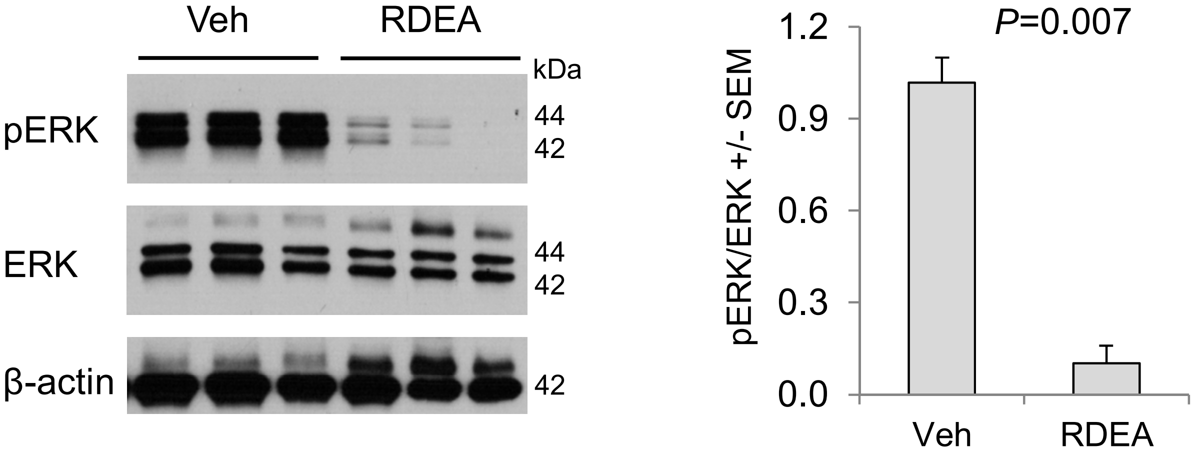

Supplementary Figure 9. Treatment with RDEA-119 inhibits ERK phosphorylation in *Tgfbr1*^{iko} ATAs. Levels of pERK were determined with western blotting assays using total protein extracted from ATAs of *Tgfbr1*^{iko} animals treated with RDEA-119 (RDEA) or vehicle dissolvent (Veh) for two weeks(*n*=6 per group). Density of each pERK immunoblot was quantified and normalized to the total ERK. Data were analyzed using unpaired t-test.

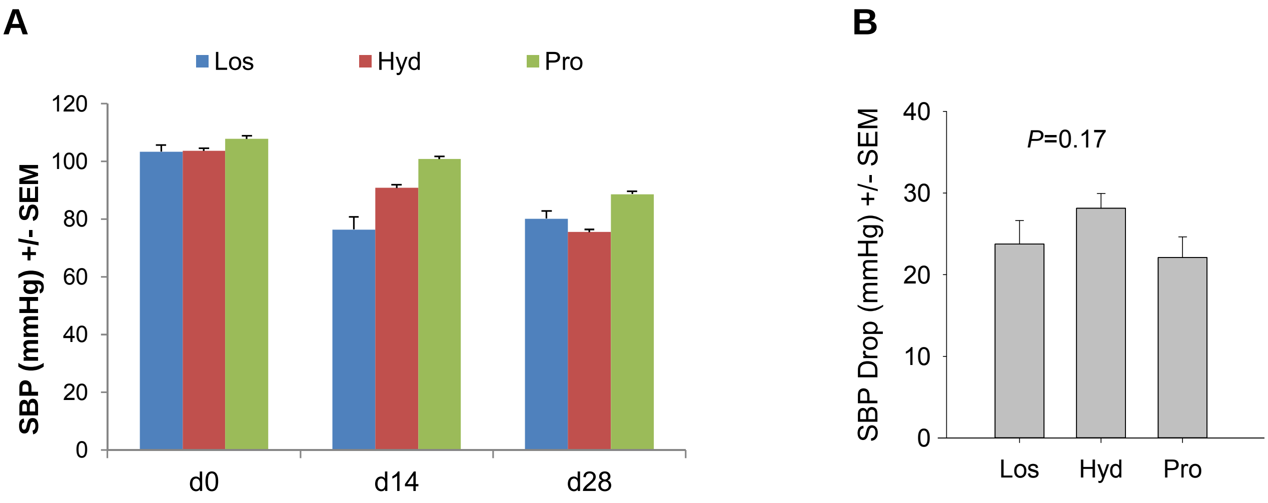

Supplementary Figure 10. Mice receiving different treatments show similar changes in the systolic blood pressure (SBP). *Tgfbr1*^{iko} mice were treated with Losartan (Los, *n*=10), Hydralazine (Hyd, *n*=12), or Propranolol (Pro, *n*=10). *P*=0.17, one-way ANOVA.

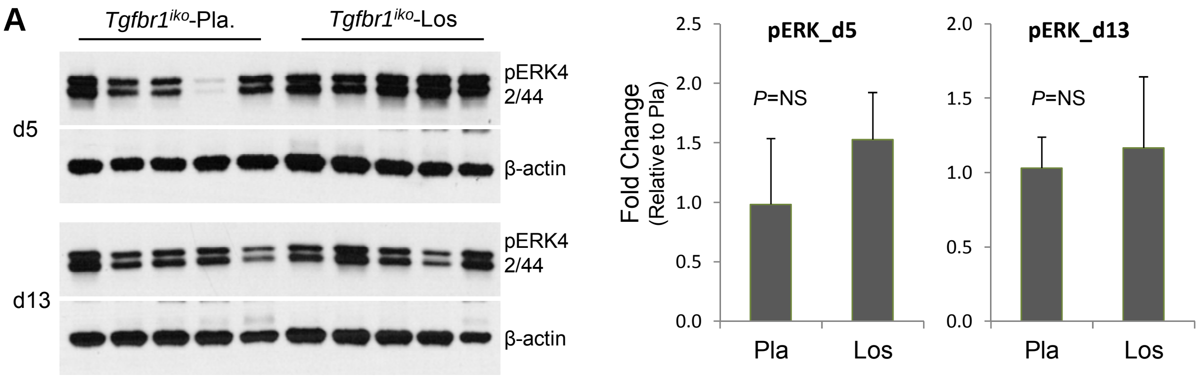

Supplementary Figure 11. The abundance of pERK in *Tgfbr1*^{iko} aortas is not impacted by treatment with losartan at the early stage. (**A**) Western blots of pERK for ATAs that were treated with placebo (Pla) or losartan (Los) at the indicated time points. (**B**) Quantification of the blots shown in panel A. Data were expressed as fold-change relative to placebo-treated controls and analyzed using the unpaired t-test.

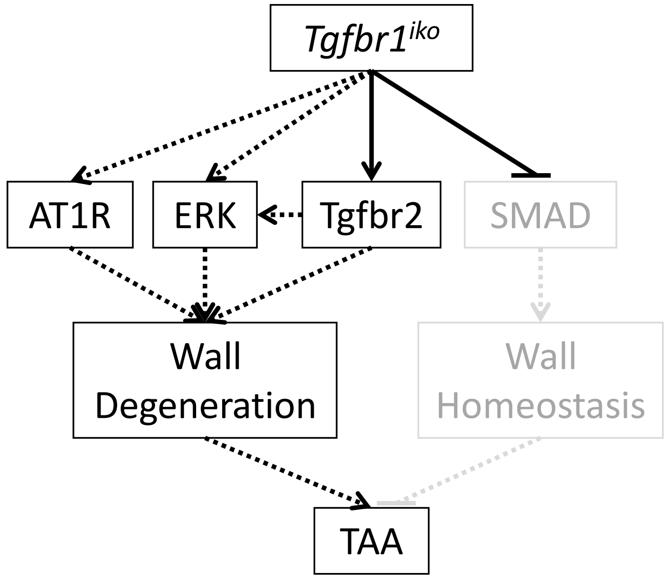

Supplementary Figure 12. A hypothetical model for the *Tgfbr1*^{iko}-driven aortic aneurysm development. Lines connect molecular and cellular events promoted by baseline TGFBR1 signaling (shown in gray) or activated as a result of *Tgfbr1*^{iko} (shown in black). Terminal arrows indicate activation or enhancement while terminal straight lines denote inhibition. Solid lines indicate cell-autonomous regulation. Dash lines indicate pathways with details to be defined.
